# Structural insights into the mechanism of adaptive ribosomal modification by *Pseudomonas* RimK

**DOI:** 10.1002/prot.26429

**Published:** 2022-10-06

**Authors:** Catriona M. A. Thompson, Richard H. Little, Clare E. M. Stevenson, David M. Lawson, Jacob G. Malone

**Affiliations:** ^1^ Department of Molecular Microbiology John Innes Centre, Norwich Research Park Norwich United Kingdom; ^2^ University of East Anglia, Norwich Research Park Norwich United Kingdom; ^3^ Department of Biochemistry and Metabolism John Innes Centre, Norwich Research Park Norwich United Kingdom

**Keywords:** crystal structure, docking modeling, protein binding, protein‐ligand interaction, protein–protein interaction, Pseudomonas

## Abstract

Bacteria are equipped with a diverse set of regulatory tools that allow them to quickly adapt to their environment. The RimK system allows for *Pseudomonas* spp. to adapt through post‐transcriptional regulation by altering the ribosomal subunit RpsF. RimK is found in a wide range of bacteria with a conserved amino acid sequence, however, the genetic context and the role of this protein is highly diverse. By solving and comparing the structures of RimK homologs from two related but functionally divergent systems, we uncovered key structural differences that likely contribute to the different activity levels of each of these homologs. Moreover, we were able to clearly resolve the active site of this protein for the first time, resolving binding of the glutamate substrate. This work advances our understanding of how subtle differences in protein sequence and structure can have profound effects on protein activity, which can in turn result in widespread mechanistic changes.

## 1 INTRODUCTION

Bacteria employ a diverse set of post‐transcriptional regulatory pathways to adapt to their environments. These range from second messenger signaling molecules such as cyclic‐di‐GMP (cdG), to small, mRNA binding proteins such as Hfq and RsmA.^1^, ^2^, ^3^, ^4^, ^5^ Recently, the directed modification of ribosomal proteins has emerged as an additional mechanism for proteome regulation. Ribosomal proteins can undergo methylation, acetylation, and the addition or removal of C‐terminal residues during the growth cycle and in response to various external stimuli.^6^, ^7^ The use of ribosomal modification to rapidly adapt to the environment is a key regulatory strategy during the exponential growth phase of bacteria,^6^ with several pairs of ligase and ribosomal subunit proteins characterized in *Escherichia coli*.^6^, ^8^

Although post‐translational ribosome protein modifications have been observed for decades, the roles of most of them in controlling cellular behavior remain elusive.^7^ For example, the modification of ribosomal 30S subunit protein S6 (RpsF) by RimK, an ATP‐dependent glutamate ligase, was first characterized in *E. coli* many years ago.^9^ However, the regulatory role of this modification was only recently determined, in members of the *Pseudomonas* genus.^6^, ^10^, ^11^ Little, Grenga, and co‐workers showed that the addition of glutamates to RpsF was not simply a post‐translational modification to increase stability or to introduce a structural change in the protein, but is in fact a complex and dynamic signaling switch that affects the translation of several key genes, including virulence factors in *Pseudomonas aeruginosa*.^10^, ^11^

Despite having a high degree of primary sequence identity (65%), the function and genetic contexts of the *Pseudomonas* and *E. coli* (RimK_EC_) RimK proteins are very different. In *Pseudomonas* spp., *rimK* is always found in an operon containing at least one other protein. This contrasts with *rimK* genes from enterobacteria, such as *rimK*_EC_, which occur independently within the genome. In opportunistic pathogens such as *P. aeruginosa*, *rimK* (*rimK*_PA_) is generally encoded together with the protease gene *rimB*, while in plant‐associated bacteria such as *P. fluorescens* and *P. syringae* the *rim* operon contains both *rimB* and the phosphodiesterase gene *rimA*.^10^, ^11^

The addition of poly‐α glutamate to the C‐terminus of RpsF has been shown to occur both when the protein is isolated independently^10^ and when it is integrated as part of the ribosomal 30S subunit.^6^ In vitro, RimK_EC_ has been shown to self‐limit the number of glutamate residues it adds to the C‐terminus of RpsF, to no more than 15 residues when overexpressed.^9^, ^12^ In contrast, RimK_PF_ lacks this inbuilt ability to limit the number of residues added to RpsF, adding an apparently unlimited number of glutamates in vitro.^10^ Interestingly, previous work with both RpsF_EC_ and RimK_EC_ has shown that these proteins can work interchangeably with their homologs in *P. fluorescens* (RimK_PF_ & RpsF_PF_). However, the addition of a defined, maximum number of glutamates to RpsF appears to be an innate characteristic of RimK_EC_.^10^, ^11^

The differences in activity seen between *Pseudomonas* RimK and RimK_EC_ are likely due to intrinsic structural differences, specific protein–protein interactions, or interactions with ligands or other small molecules. It seems likely that this change is related to the co‐evolution of *Pseudomonas* RimK with the protease RimB, which is able to cleave the additional glutamates to leave a truncated RpsF tail of around four residues.^11^ Our previous work identified an interaction between RimK_PF_ and the *P. fluorescens* RimB (RimB_PF_) protein,^10^ however, the specific nature of this interaction is currently poorly understood.^11^

Structural studies of RimK to date have been limited to RimK_EC_, which has been resolved as a tetramer.^12^, ^13^ At this stage, however, the structural biology of the *Pseudomonas* Rim system is largely unstudied. Here, we solve the structures of two *Pseudomonas* RimK proteins and use them to shed new light on the mechanism of protein function. A detailed comparison of these proteins allows us to identify key structural factors, such as the hitherto unknown location of glutamate binding, which help to explain the differences in enzyme activity and protein regulation seen for different RimK variants. Finally, we use the structural differences between these proteins to identify a potential RimB‐RimK interaction site, which is specific to *Pseudomonas* RimK and allows for the regulation of enzyme activity.

## 2 MATERIALS AND METHODS

### 2.1 Plasmids and strains used

Strains, plasmids, and primers used in this study are listed in Table S1. Briefly, the open reading frames of *rimK* from *E. coli*, *P. aeruginosa* PAO1, and *P. syringae* pv. *tomato* DC3000 were cloned into pET42b(+) with NdeI/XhoI and the NdeI/HindIII or NdeI/XhoI sites of pET29a (*E. coli*, PA01, and DC3000 respectively) to produce expression vectors with C‐terminal hexahistidine tags. BL21 (DE3) pLysS (Promega) cells were transformed with the resulting vectors for protein purification.

### 2.2 Recombinant protein expression

BL21 (DE3) pLysS cultures containing the desired overexpression vectors were grown overnight at 37°C shaking at 250 rpm. Overnight cultures were used as a 1% inoculum for 1 L LB cultures, which were grown at 30°C, shaking at 220 rpm until reaching the mid‐log phase (OD_600_ = 0.6). Cells were induced with 1 mM IPTG and grown for a further 2 h. Cells were harvested at 5000 x*g*, 4°C, 10 min, and then frozen at –80°C.

Cells were resuspended in sample buffer (20 mM HEPES, 250 mM NaCl, 10 mM MgCl_2_ 2.5% glycerol pH 6.8) and lysed using an Avestin cell disruptor. The insoluble fraction was removed by centrifugation at 33000 x*g* for 40 min and the soluble fraction was loaded onto a pre‐equilibrated 1 ml His‐Trap Excel column (Cytiva). The column was washed in 8% elution buffer (20 mM HEPES, 250 mM NaCl, 10 mM MgCl_2_ 1 M imidazole, 2.5% glycerol pH 6.8) and eluted over a gradient of 8%–100% elution buffer over 10 ml. Fractions were analyzed by SDS‐PAGE and buffer exchange was carried out using Zeba Desalt spin columns (Thermo Scientific) as per the manufacturer's protocol.

### 2.3 Crystallography

Crystallization screens were set up in sitting‐drop vapor diffusion formats in MRC2 96‐well crystallization plates with 0.3 μl precipitant solution and 0.3 μl protein and incubated at 293 K. After optimization of initial hits, suitable crystals were cryoprotected and mounted in Litholoops (Molecular Dimensions) before flash‐cooling by plunging into liquid nitrogen. X‐ray data were recorded on beamline I03 at the Diamond Light Source (Oxfordshire, United Kingdom) using a Pilatus3 6 M hybrid photon counting detector (Dectris), with crystals maintained at 100 K by a Cryojet cryocooler (Oxford Instruments). Diffraction data were integrated and scaled using XDS^14^ or DIALS^15^ via the XIA2 expert system^16^ then merged using AIMLESS.^17^ Data collection statistics are summarized in Table 1. The majority of the downstream analysis was performed through the CCP4i2 graphical user interface.^18^

**TABLE 1:** Summary of RimK X‐ray data and model parameters.

Protein source	*P. aeruginosa* PA01	*P. syringae* DC3000
Data collection		
Diamond light source beamline	I03	I03
Wavelength (Å)	0.976	0.976
Detector	Pilatus3 6 M	Pilatus3 6 M
Resolution range (Å)	44.45–2.40 (2.44–2.40)	49.80–2.90 (2.95–2.90)
Space group	*P*2_1_	*P*1
Cell parameters (Å/°)	*a* = 66.9, *b* = 153.6, *c* = 138.5, *β* = 102.4	*a* = 95.9, *b* = 96.1, *c* = 157.0, *α* = 88.9, *β* = 84.4, *γ* = 90.0
Total no. of measured intensities	733 468 (35144)	419 535 (19555)
Unique reflections	105 678 (5171)	122 285 (6015)
Multiplicity	6.9 (6.8)	3.4 (3.3)
Mean *I*/σ(*I*)	10.9 (0.9)	5.8 (1.1)
Completeness (%)	99.3 (98.8)	98.9 (98.6)
*R*_merge_ ^a^	0.108 (2.049)	0.161 (0.976)
*R*_meas_ ^b^	0.116 (2.219)	0.192 (1.174)
*CC*_½_ ^c^	0.999 (0.438)	0.983 (0.430)
Wilson *B* value (Å^2^)	59.7	47.2
Refinement		
Resolution range (Å)	44.45–2.40 (2.46–2.40)	49.80–2.90 (2.98–2.90)
Reflections: working/free^d^	100 447/5200	116 340/5892
*R*_work_ ^e^	0.204 (0.341)	0.237 (0.361)
*R*_free_ ^e^	0.228 (0.336)	0.248 (0.386)
Ramachandran plot: favored/allowed/disallowed^f^ (%)	97.1/2.8/0.1	95.4/4.1/0.5
R.m.s. bond distance deviation (Å)	0.008	0.004
R.m.s. bond angle deviation (°)	1.46	1.30
Protein (chains/residues/av. *B* [Å^2^])	8/2295/76	16/4587/67
Poly‐Glu (chains/residues/av. *B* [Å^2^])	8/57/111	−/−/−
ADP (molecules/av. *B* (Å^2^)/RSCC)^g^	8/103/0.74–0.94	11/86/0.72–0.87
Water (molecules/av. *B* [Å^2^])	102/55	−/−
Overall av. *B* (Å^2^)	77	67
PDB accession code	7QYR	7QYS

*Note*: Values in parentheses are for the outer resolution shell.

^a^ *R*_merge_ = ∑_*hkl*_ ∑_*i*_ |*I*_*i*_ *(hkl)* ‐ ⟨*I(hkl)*⟩|/∑_*hkl*_ ∑_*i*_ *I*_*i*_ *(hkl)*.

^b^ *R*_meas_ = ∑_*hkl*_ [*N*/(*N* ‐ 1)]^1/2^ × ∑_*i*_ |*I*_*i*_ *(hkl)* ‐ ⟨*I(hkl)*⟩|/∑_*hkl*_ ∑_*i*_ *I*_*i*_ *(hkl)*, where *I*_*i*_ *(hkl)* is the *i*th observation of reflection *hkl*, ⟨*I(hkl)*⟩ is the weighted average intensity for all observations *i* of reflection *hkl* and *N* is the number of observations of reflection *hkl*.

^c^ *CC*_½_ is the correlation coefficient between symmetry equivalent intensities from random halves of the dataset.

^d^ The data set was split into “working” and “free” sets consisting of 95% and 5% of the data respectively. The free set was not used for refinement.

^e^ The R‐factors *R*_work_ and *R*_free_ are calculated as follows: *R* = ∑(|*F*_obs_ ‐ *F*_calc_|)/∑|*F*_obs_|, where *F*_obs_ and *F*_calc_ are the observed and calculated structure factor amplitudes, respectively.

^f^ As calculated using MolProbity.^30^

^g^ Real Space Correlation Coefficient as calculated by the PDB Validation Server (https://validate‐rcsb‐1.wwpdb.org).

*P. aeruginosa* PAO1 RimK (RimK_PA_) in purification buffer was placed into crystallization trials at a concentration of 17 mg/ml. Optimized crystals grew in 0.2 M potassium sodium tartrate tetrahydrate, 0.1 M bis‐tris propane, pH 7.5, 20% wt/vol PEG 3350 (PACT Premier Screen, Molecular Dimensions) and were cryoprotected in the crystallization solution supplemented with 20% PEG3350, 20% ethylene glycol, 90 mM NPS, 100 mM MES pH 6.5. X‐ray data were processed to a resolution of 2.4 Å in space group *P*2_1_ with cell parameters *a* = 66.9 Å, *b* = 153.6 Å, *c* = 138.5 Å, *β* = 102.4°. A template for molecular replacement was prepared from the RimK_EC_ structure (PDB: 4IWK), which has a 65.5% sequence identity to RimK_PA_. The structure was solved by molecular replacement with PHASER,^19^ which located eight copies of the template in the asymmetric unit (ASU), corresponding to an approximate solvent content of 52%, and gave *R*_work_ and *R*_free_ values of 0.308 and 0.345, respectively, to 2.4 Å resolution after jelly body refinement with REFMAC5.^20^ A complete rebuild in BUCCANEER^21^ followed by several iterations of model editing in COOT^22^ and restrained refinement in REFMAC5 yielded the final model with *R*_work_ and *R*_free_ values of 0.203 and 0.228, respectively, to 2.4 Å resolution (see Table 1). Weak density was apparent for nucleotides in all of the active sites, but this was only sufficient to resolve the adenine and ribose rings of ATP/ADP. Additional regions of electron density adjacent to the active sites of seven of the subunits and trapped in a crystal contact were interpreted as poly‐α‐glutamate. In the case of the latter region, this formed a four‐stranded mixed β‐sheet.

*P. syringae* DC3000 RimK (RimK_PS_), in purification buffer was placed into crystallization trials at 3.7 mg/ml. Optimized crystals grew in a solution containing: 0.2 M calcium chloride dihydrate, 0.1 M HEPES pH 7.0, 20% wt/vol PEG6000 and were cryoprotected in the crystallization solution of 18% PEG 3350, 18% ethylene glycol, 80 mM bis tris pH 6.5, 180 mM sodium citrate, 1 mM ATP, 1 mM CdG. X‐ray data were processed to a resolution of 2.9 Å in space group *P*1 with cell parameters *a* = 95.9 Å, *b* = 96.1 Å, *c* = 157.0 Å, *α* = 88.9°, *β* = 84.4°, *γ* = 90.0°. A template for molecular replacement was prepared from the RimK_EC_ structure (PDB: 4IWK), which has 64.5% sequence identity to RimK_PS_. The structure was solved by molecular replacement with PHASER, which located 16 copies of the template in the asymmetric unit (ASU), corresponding to an approximate solvent content of 53%, and gave *R*_work_ and *R*_free_ values of 0.271 and 0.309, respectively, to 2.9 Å resolution after jelly body refinement with REFMAC5. A complete rebuild in BUCCANEER, followed by several iterations of model editing in COOT and restrained refinement in REFMAC5 yielded the final model with *R*_work_ and *R*_free_ values of 0.238 and 0.248, respectively, to 2.9 Å resolution (see Table 1). Weak density was apparent for the nucleotide in 11 of the active sites, which was interpreted as ADP.

### 2.4 Linked pyruvate kinase/lactate dehydrogenase (PK/LDH) ATPase activity assays

ATPase activity was measured indirectly by monitoring NADH oxidation in a microplate spectrophotometer (BioTek instruments) at 25°C. The reaction buffer consisted of 100 mM TRIS–HCl (pH 9.0) and 20 mM MgSO_4_. Each reaction contained 0.4 mM NADH, 0.8 mM phosphoenolpyruvic acid, 0.7 μl PK/LDH (Merck) and was initiated by the addition of 10 μl ATP for ATP titration experiments or 1 μl of 200 mM ATP for assays measuring the response of RimK ATPase activity to RimB. Protein concentrations were as shown in the figure legends. Enzyme kinetics were determined by measuring absorbance at 340 nm at 1‐min intervals. Kinetic parameters were calculated by plotting the specific activity of the enzyme (nmol ATP hydrolyzed/min/mg protein) versus ATP (0–2 mM) or RimB (0–1.2 mM) concentration and by fitting the non‐linear enzyme kinetics model (Michaelis–Menten) in Graph‐Pad Prism.

### 2.5 HADDOCK docking

Docking was performed using the HADDOCK 2.4.1 server.^23^ The monomeric subunits of RimK_PA_ and RimK_PS_ with the active residues chosen being the non‐conserved and partially conserved residues for each protein compared to RimK_EC_. The active residues for RimK_PA_ were: 5, 9, 10, 11, 15, 16, 22, 23, 24, 28, 29, 30, 31, 32, 36, 37, 42, 43, 44, 45, 46, 47, 51, 53, 54, 56, 57, 67, 69, 74, 76, 83, 85, 86, 97, 104, 108, 110, 113, 119, 126, 127, 131, 140, 143, 154, 157, 158, 164, 165, 171, 172, 173, 175, 184, 186, 191, 196, 198, 200, 201, 202, 204, 206, 207, 209, 219, 222, 223, 224, 228, 231, 232, 235, 239, 241, 243, 252, 263, 272, 276, 282, 284, 285, 286, 288, 289, 290, 291, 292, 293, 294, 295, 296, 297, 298.

## 3 RESULTS

### 3.1 Crystallographic structures of *Pseudomonas* RimK variants

RimK variants from *Pseudomonas* species are predicted to be broadly structurally similar to RimK_EC_. However, we hypothesized that some of the observed functional differences between RimK proteins may be explained by molecular‐level structural differences. To examine this, we determined the structures of RimK homologs from two different *Pseudomonas* species, *P. syringae* pv. *tomato* DC3000 (RimK_PS_) and *P. aeruginosa* PAO1 (RimK_PA_), as representative models (Table 1). Both proteins were purified from an *E. coli* BL21 expression host. RimK_PA_ was crystalized in the absence of its substrates; ATP, cdG, and glutamate whereas RimK_PS_ was crystalized with both ATP and cdG present in the cryoprotectant. Both RimK_PS_ and RimK_PA_ were resolved as tetramers with at least one substrate bound. ADP and glutamate, of presumed cellular origin, were resolved in the RimK_PA_ structure whereas ADP alone was resolved in the RimK_PS_ structure.

Previous work has shown that RimK_PF_ and RimK_PS_ are biochemically interchangeable, having similar ATPase activity levels and the ability to add an unlimited number of glutamate residues to RpsF, suggesting they have similar modes of action in vivo.^10^ Interestingly, RimK_EC_ and RimK_PA_ both appear to function quite differently from the others, with RimK_EC_ activity being self‐limiting in the number of additional glutamate residues it adds to RpsF and RimK_PA_ displaying an intermediate activity between RimK_PS_ and RimK_EC_. As we were unable to resolve cdG in either of our crystal structures, we examined the stimulation of ATPase activity by cdG in both RimK_PA_ and RimK_PS_ (Figure S1). As previously observed by Grenga and colleagues^11^ the addition of cdG stimulated ATPase activity for both proteins, with the addition of cyclic‐di‐AMP showing no stimulation, suggesting this is a specific response.

**FIGURE 1:**
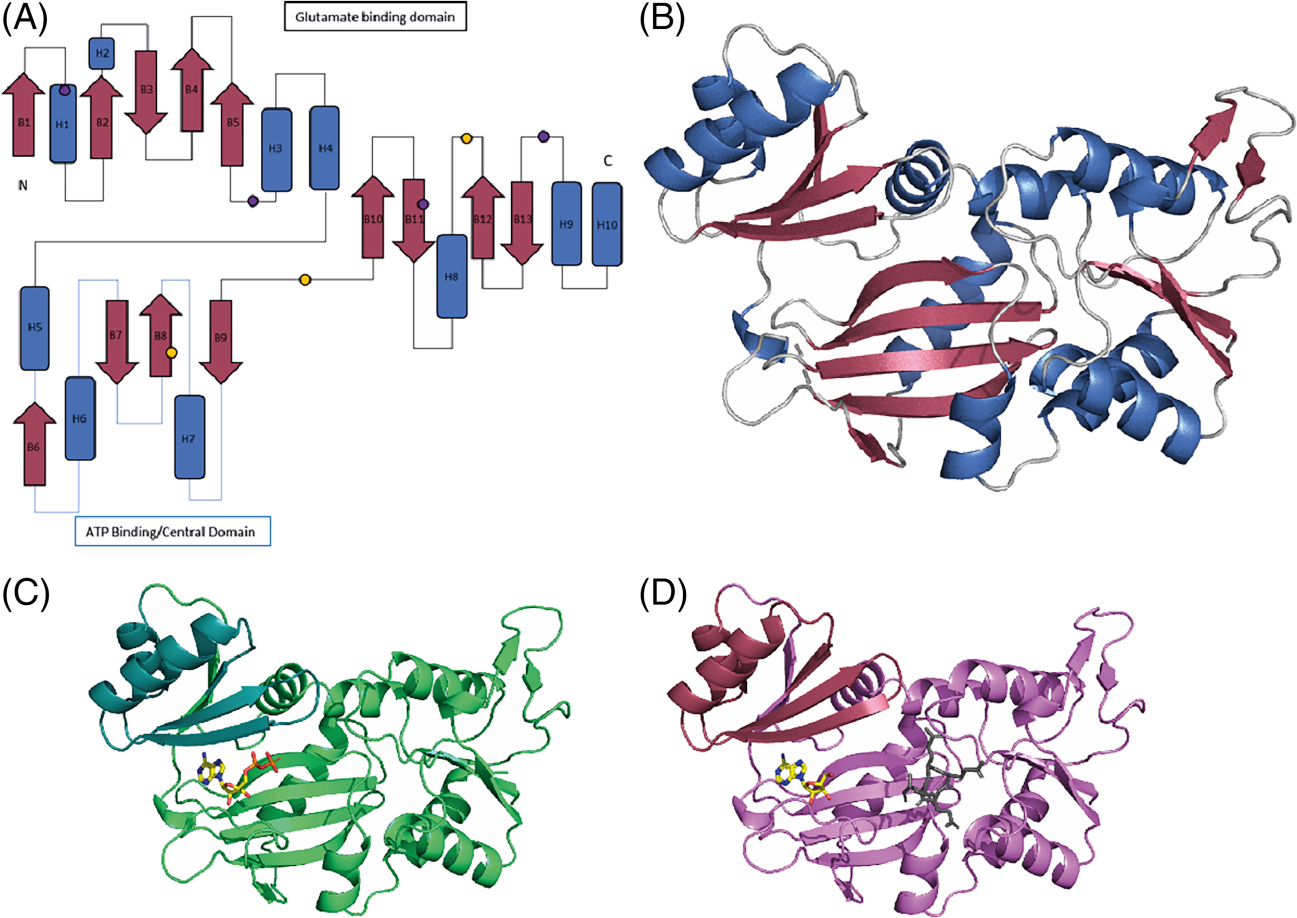
Monomeric structures of RimK homologues from *Pseudomonas* spp. (A) Example schematic of the secondary structure of RimK_PS_ Chain A, with α‐helices shown in blue and βsheets shown in red. The central ATP binding domain is highlighted with blue linkers with the approximate locations of key residues for both the ATP (yellow) and glutamate (purple) active sites are shown as circles. (B) Cartoon representation of RimK_PS_ Chain A with α‐helices shown in blue and β‐sheets shown in red, with the central ATP binding domain highlighted in the blue dashed box. Cartoon representations of monomeric units (chain A) of RimK_PS_ (C) and RimK_PA_ (D) are shown with the upper ATP binding region shown in teal and raspberry respectively.

Each individual RimK unit has a classical ATP‐grasp fold with an elongated shape of approximately 60 x 35 x 30 Å. The core of the protein is made up of four antiparallel β‐sheets (B10‐B13) that are encased by α‐helices H3 and H4 on one side and by H7 and H8 on the opposite side. This core domain contains the main predicted glutamate binding residues^12^ and is connected to the ATP binding domain by a flexible linker (Figure 1A,B). The ATP binding domain contains two α‐helices (H5 and H6) and four antiparallel β‐sheets (B6‐9) (highlighted in Figure 1B). The ATP binding domain, despite having a highly conserved secondary structure, is the most divergent domain of the protein with regard to tertiary structure (Figure 1B). This divergence could be due to many factors but also suggests that this domain may be flexible. The ATP binding domain then leads onto a set of α‐helices that complete the active site. The final loops and remaining β‐sheets loop round to terminate next to the N‐terminus.

**FIGURE 2:**
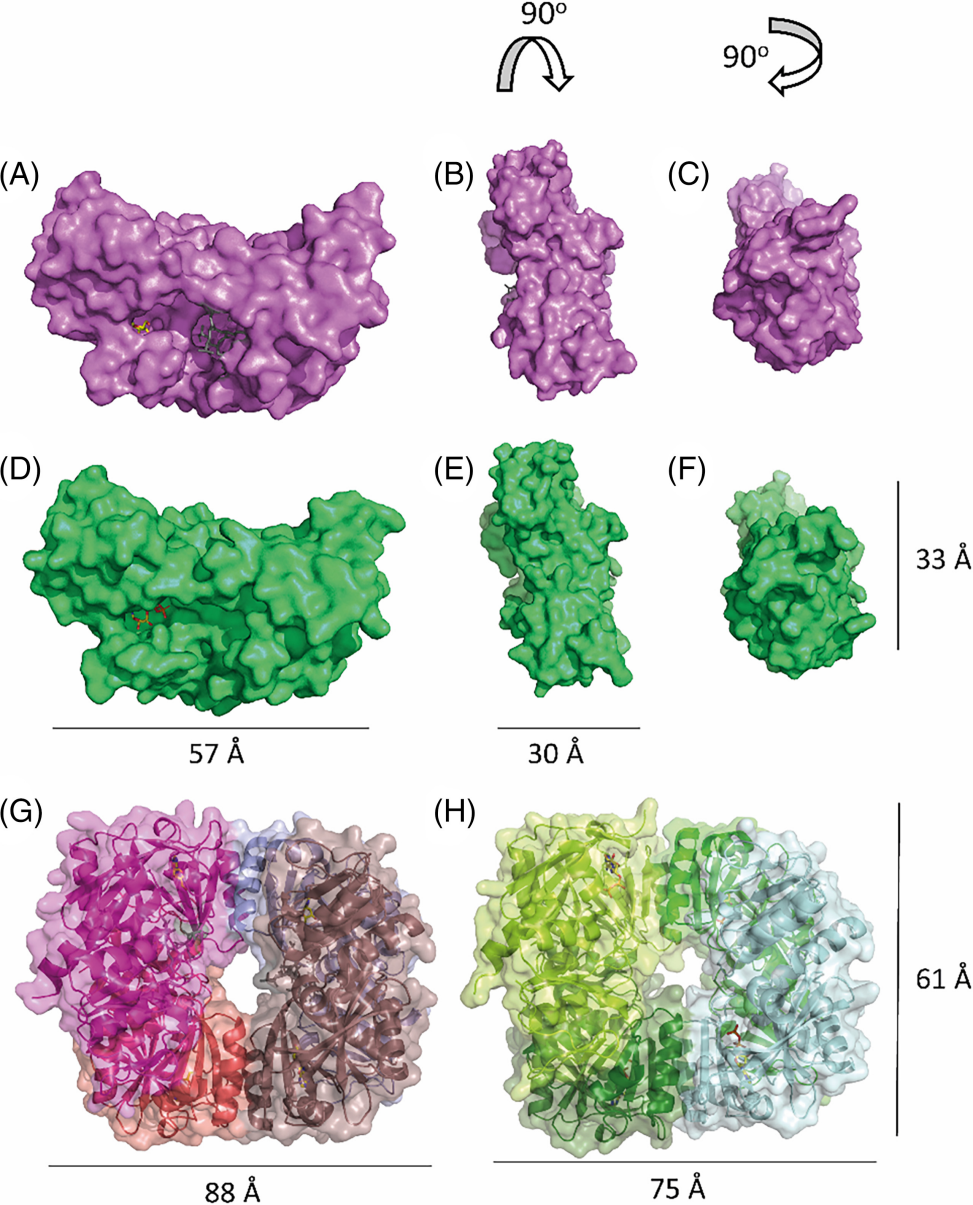
Surface structures of the monomeric and tetrameric units of each of the RimK homologues. Surface representation of high‐resolution structures and measurements of RimK_PA_ (A–C) and RimK_PS_ (D–F) for the monomeric units of each of the proteins (chain A). Measurements of the tetrameric subunits for RimK_PA_ (G) and RimK_PS_ (H) are shown for two dimensions.

Although the RimK structures were resolved as tetramers, when the monomeric unit of each of the RimK homologs was examined both the secondary (Figure 1A) and tertiary (Figure 2) structures initially appeared to be very similar. RimK_PA_ has a slightly more closed tertiary structure in the center of the protein, leading to a more occluded active site that contains the predicted glutamate binding pocket. This occlusion is due to several factors, including the addition of a small section of β‐sheet in the core of the protein as well as the divergence of H2. There is a larger loop (Figure 1C,D, highlighted by a black arrow) present at the bottom of the protein active site that is less prominent in RimK_PS_. Loops within proteins can be highly variable and flexible, however, taken together these data support the observation of a smaller active site cleft in RimK_PA_.

Tetramer formation is consistent both with previously solved RimK homologs^13^ and our current models for *Pseudomonas* RimK function.^10^, ^11^ It is possible that conformational changes may occur to the monomeric subunits when oligomerization occurs, potentially leading to the closure of the glutamate binding pocket. For both resolved *Pseudomonas* tetramers the ATP binding domain interacts with the unstructured N‐terminal loops of another protein subunit to form a dimer. This dimer then interacts with a second dimer to form a symmetrical tetrameric unit, with the ATP binding interface on the periphery of the complex facing inward, and the glutamate binding region in the core of the tetramer (Figure 2G,H). Interestingly, each of the monomeric subunits within the tetramers for both *P. aeruginosa* and *P. syringae* have the same secondary and tertiary structure, which is in contrast to the recent *E. coli* tetramer structure that contained two distinct conformations of each monomer.^13^ Although structurally similar, the RimK_EC_ and *Pseudomonas* RimK proteins have distinctly different glutamate ligase activities.^10^, ^11^ Therefore, it is possible these differences are linked to small structural differences between the proteins either in their monomeric or tetrameric forms.

### 3.2 Small structural variations in the ATP binding domain lead to large activity changes

**FIGURE 3:**
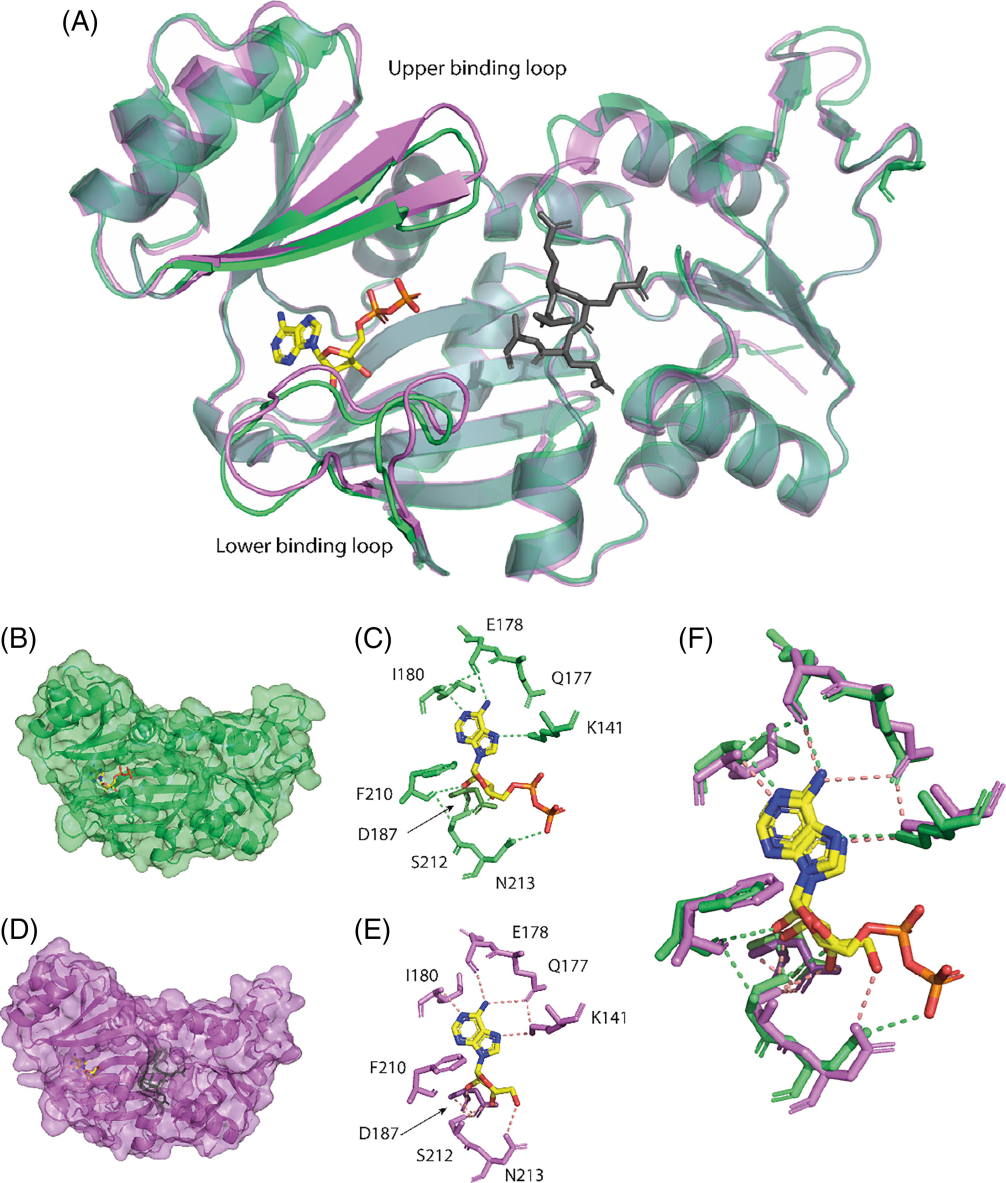
ATP/ADP binding pocket of both RimK_PS_ and RimK_PA_ show a conserved binding motif between the two proteins. (A) Cartoon overlay of RimK_PS_ (green) and RimK_PA_ (pink) with ATP (yellow) and glutamate (gray) with the upper and lower ATP binding loops highlighted (B) Cartoon and surface representation of RimK_PS_ with ADP highlighted in its binding pocket with yellow sticks. (C) Electrostatic interactions between side chains and ADP molecule that were identified by crystallization. (D) Cartoon and surface representation of RimK_PA_ with ADP highlighted in yellow and glutamate residues highlighted in gray. (E) Electrostatic interactions between side chains and ADP molecule that were identified by crystallization for RimK_PA_. (F) Overlay of the ADP binding residues and interactions for RimK_PS_ (green) and RimK_PA_ (pink).

RimK belongs to the ATP‐dependent carboxylate‐amine ligase superfamily of enzymes^13^, ^24^ which includes other members which can add amino acids to the C‐terminus of their target proteins.^25^ The glutamate binding domain of each RimK subunit is linked to the ATP‐binding central domain by a flexible linker region, which is likely to allow the protein a range of motion to enable it to act like a classical “clam shaped” enzyme. This is thought to enable RimK to bring the bound ATP molecule to the active site, allowing it to energize the ligation of glutamate residues, as seen in similar amino acid ligases.^25^, ^26^ Within the RimK_PS_ and RimK_PA_ structures, the bound nucleotide was interpreted as ADP rather than ATP in the predicted ATP binding site, potentially due to a dephosphorylation event prior to crystallization. The position of the ADP molecule within our RimK crystal structures is highly conserved within both RimK proteins and with other ATP‐dependent amino acid ligases, such as LysX^25^, ^26^ with the nucleotide molecule positioned in the cleft between the two domains (Figure 3A,B, and D). For RimK_PS_ and RimK_PA_, the ADP molecule interacts with a core set of six side chains: K141, E178, I180, F210, S212, and N213 (Figure 3B and D). However, there is a degree of variation between the additional side chains that make up the binding site in each case (Figure 3E).

Previous work by Zhao and colleagues^12^ identified that the RimK_Ec_ protein contained several important loops that made up a section of the active site, including two which are part of the ATP binding domain and contain residues that directly interact with the ADP/ATP substrate. All *Pseudomonas* RimK structures contain the lower of these two binding loops, which was found to be in a similar orientation in both RimK_PS_ and RimK_PA_ (highlighted in Figure 3A) however, the positioning of these residues leads to slight alterations in the occupied space of the loop within the active site. More substantial differences were observed in the upper ATP binding loop (highlighted in Figure 3A), with notable changes in the β‐sheet formations. Within the upper loops, the β‐sheet of RimK_PS_ is shorter than that of RimK_PA,_ potentially allowing more flexibility in the loop. More interestingly, a distinct difference was observed between the residues that make up the upper and lower ATP binding loops of RimK_EC_ and RimK_PS/PA_ despite several shared residues being present within the homologues. This may hint toward a mechanistic difference between the proteins, which accords with previously obtained biochemical data.^10^ Intriguingly, a closer examination of the RimK_PA_ structure highlighted a set of residues that were unresolved in several chains, and that correspond to the lower substrate binding loop. This suggests that this lower loop could be flexible and move with the ATP/ADP molecule, which is also found in slightly different orientations in each chain.

**FIGURE 4:**
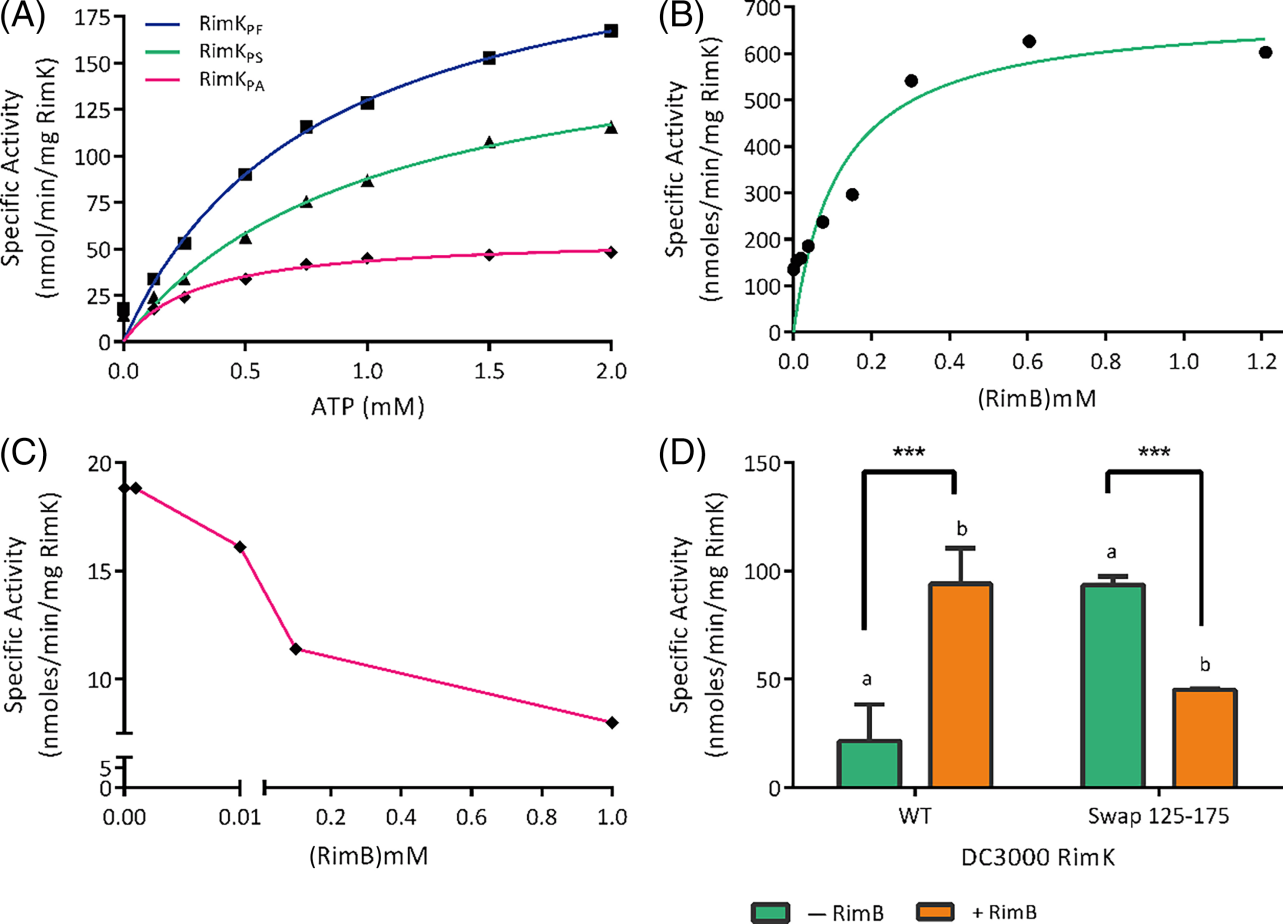
ATPase activity varies between RimK_PA_ and RimK_PS/PF_ and is influenced by the addition of RimB. (A) ATPase assays were carried out on freshly purified RimK proteins, which showed a lower rate of ATPase activity for RimK_PA_ (*V*_max_ = 57 nm/min/mg, *K*_m_ = 0.3 mM) compared with both RimK_PF_ (*V*_max_ = 234 nm/min/mg, *K*_m_ = 0.8 mM) and RimK_PS_ (*V*_max_ = 176 nm/min/mg, *K*_m_ = 1 mM). Individual points represent absolute data points with the non‐linear regression fit shown as a solid line. RimK was present at a concentration of 1.5 μM in each case. (B) The addition of RimB_PS_ increases RimK_PS_ ATPase activity rate (*V*_max_ = 692 nm/min/mg, *K*_m_ = 0.12 mM). (C) Conversely, RimB_PA_ leads to a reduction in the rate of ATPase activity when incubated with RimK_PA_, with RimK present at a concentration of 3.6 μM, ATP present at 200 mM and RimB present at a concentration of 0–1.2 mM in each experimental condition. (D) ATPase results for RimK_Ps_‐WT and RimK_Ps_ swap (AAs 125–175 from RimK_Pa_) with (orange) and without (green) the presence of RimB_Ps_ at a concentration of 2 mM. A two‐way ANOVA indicated significance for the interaction (*p* < .0001), with multiple comparisons showing significant differences between RimK_Ps_‐WT and RimK_Ps_‐Swap both without (a, *p* < .01) and with RimB (b, *p* < .01). A significant effect of RimB was seen for both proteins (***, *p* < .001, Sidak's multiple comparisons test)

The divergence in the ATP binding sites of RimK_PS_ and RimK_PA_ at first appears to be quite modest. However, these differences are functionally significant. Previous biochemical analysis of the ATPase activities of RimK_PF_ and RimK_PS_ showed that these proteins are functionally interchangeable in their ability to hydrolyze ATP,^10^ with similar ATPase rates observed (Figure 4A). However, we observed significant differences between the ATPase activity of RimK_PA_ and the other two *Pseudomonas* RimK homologs, with RimK_PA_ showing a significantly reduced *V*_max_ compared with both RimK_PS_ and RimK_PF_ (Figure 4). These differences in ATPase activity are likely due to intrinsic structural differences between the RimK_PS/PF_ and RimK_PA_ proteins.

The interaction of RimK with its' binding partner RimB has previously been shown in *P. fluorescens* to dramatically increase the rate of ATP hydrolysis.^11^ RimK_PF_ and RimK_PS_ are genetically similar. Consequently, and as expected the addition of RimB_PS_ stimulated RimK_PS_ ATPase activity in a concentration dependent manner (Figure 4B). Conversely, the addition of increasing concentrations of RimB_PA_ led to a reduction in RimK_PA_ ATPase activity, suggesting that there are important, intrinsic differences between the responses of these two proteins to their corresponding binding partners. Therefore, although the structural differences in the ATP binding site are small, these differences are linked to large changes in both the extent and regulation of enzymatic activity. This becomes particularly noticeable upon the addition of RimB. One possibility is that the interaction between RimK and RimB leads to a conformational change in the protein resulting in a change in the dimensions of the active site. It is possible that the addition of RimB closes the active site of RimK, which in RimK_PS_ may result in more optimal conformation for the ligation of glutamates but may hinder this activity in RimK_PA._

While there are a number of residue differences between RimK_PS_ and RimK_PA_, the most divergent region between the two proteins is in the ATP binding loop (AAs 125–175). This loop is therefore a prime candidate for the hypothesized structural differences that result in divergent regulation for RimK_PS_ compared to RimK_PA_ in the presence of RimB. To test this, a chimeric protein based on RimK_PS_ but with residues 125–175 changed for the PA01 residues (RimK_PS_‐Swap 125–175). Interestingly, the basal activity of RimK_PS‐_swap 125–275 in the absence of RimB was higher than that of the WT. When incubated with 2 mM RimB, the ATPase activity of WT RimK_PS_ was strongly stimulated. However, the opposite was true for the RimK_PS_‐Swap 125–175 chimera, which showed clear suppression of ATPase activity upon RimB addition. This supports the hypothesis that structural differences between the RimK_PS_ and RimK_PA_ ATP binding sites are responsible for the divergent RimB regulation seen for these proteins and locates the causal residues in the 125–175 ATP binding loop.

### 3.3 Glutamate binds to a highly conserved set of residues in the RimK active site

While the glutamate ligase activity of RimK_EC_ is well established,^27^ the specific binding site for glutamate in the protein's active site has remained elusive. Previous structural work with RimK_EC_ predicted that the glutamate binding site consisted of 16 large, charged residues^12^ . However, it was not possible to resolve glutamate chains in either of the RimK_EC_ crystal structures.^12^, ^13^ In this study, we were able to resolve a peptide fitting the profile of poly‐α‐glutamate chains of either three or five amino acids long, in the active sites of seven out of eight of RimK_PA_ monomer subunits. This allowed us to confirm the active site location, which had previously only been predicted.^12^

**FIGURE 5:**
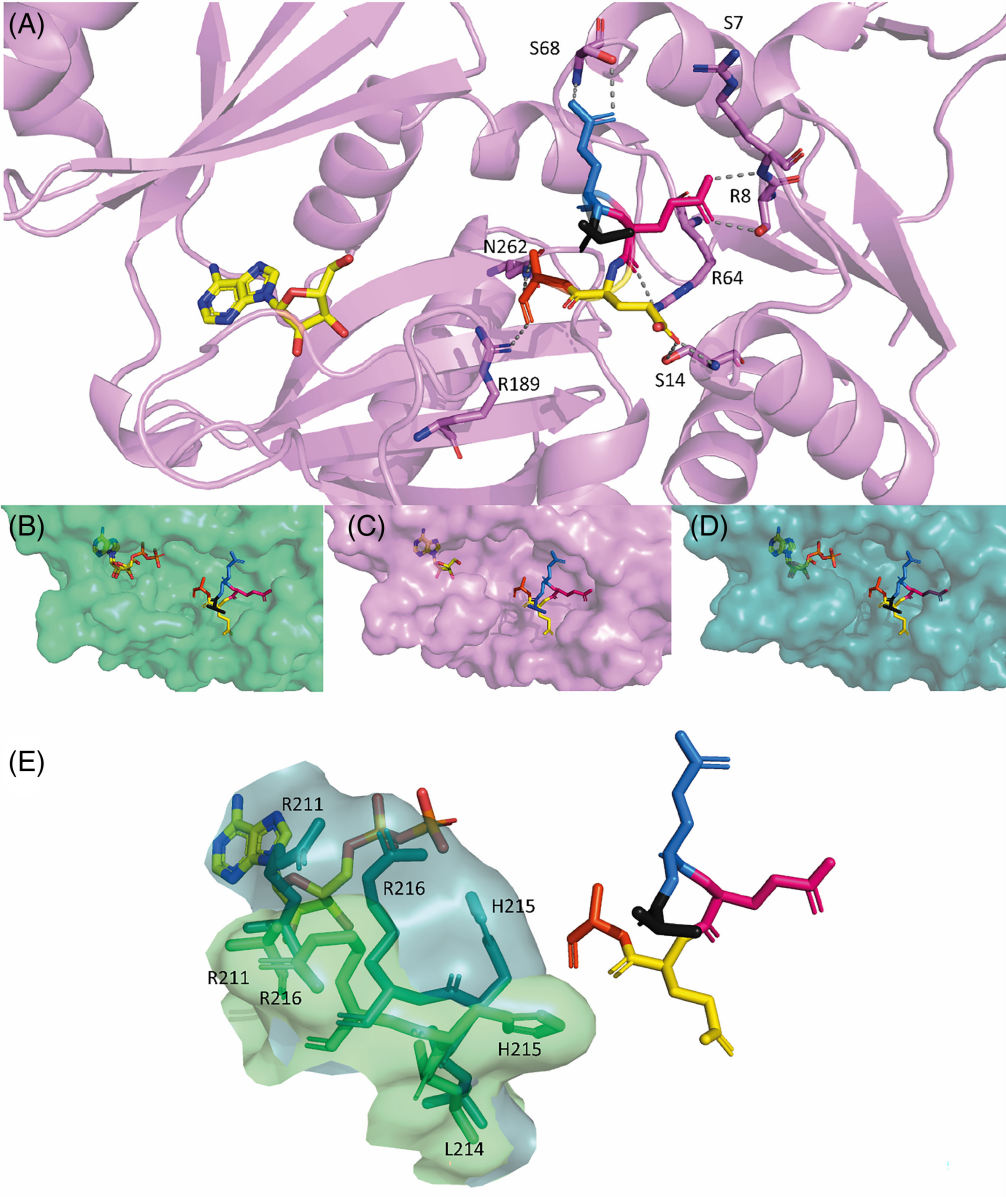
Cartoon and surface representations of the glutamate binding site in RimK showing key residues. A representation of the resolved glutamate chain (colored sticks E1‐5 in blue to orange) from RimK_PA_ chain A is shown (A) with interacting residues shown in gray. Surface and stick representation of the binding pockets of RimK_PS_ (B), RimK_PA_ (C) and RimK_EC_ (D) highlighting the differences in the glutamate and ATP binding pockets, with glutamate (colored) and ATP/ADP (yellow) shown as sticks. (E) ATP binding loop residues that contribute to the changes in the glutamate binding loop between RimK_PS_ (green) and RimK_EC_ (teal) with glutamate shown as sticks.

RimK_PA_ chain A was examined in more detail as it was the most complete monomeric unit with a fully resolved structure as well as both ADP and glutamate bound. From the high‐resolution structure, the first resolved glutamate residue (E1) of the chain does not interact with any known side chains. However, interactions were observed between each of the other four glutamate residues and the RimK_PA_ active site. Interactions between side chains and residues less than 3 Å away were examined, confirming that the second resolved glutamate residue (E2) interacts with S68, E3 interacts with S7, R8 and R64, E4 interacts with S14 and finally E5 interacts with N262 and R189 (Figure 5A). Unsurprisingly, many these interacting residues are arginines, which are positively charged and therefore can easily interact with the negatively charged glutamate residues. These interacting residues are similar to those predicted by Zhao and colleagues in their recent paper.^12^

Next, the 5E chain from the RimK_PA_ structure was modeled into the active sites of RimK_EC_ and RimK_PS_ to gain insights into the functional differences between them. Although there is a high degree of sequence similarity between the active sites of the different RimK proteins, clear tertiary structure differences were present within their binding sites (Figure 5B–D), with the RimK_PS_ binding site appearing significantly more open than that of RimK_EC_.

To examine these differences in more detail, the orientation of the side chains in the lower substrate binding loop were compared between RimK_PS_ and RimK_EC_. There are very few differences between the side chain orientations of the active site, however three key residues appear to contribute to a significant change in the active site dimensions (Figure 5E). The residues H215, R216 and R211 are oriented in opposite directions in RimK_EC_ and RimK_PS_, with all three pointing towards the active site in RimK_EC_, thus reducing the size of the active site cleft and encasing the ATP/ADP binding site. This difference in active site structure may give some insight as to why the glutamate ligation rates vary between the RimK variants.

### 3.4 Structural divergence between *E. coli* and *Pseudomonas* RimK provides insight into their mechanistic differences

Although superficially similar in their tertiary structure, the mechanisms by which RimK_EC_, RimK_PA_ and RimK_PS_ act both in vitro and in vivo are very different. The main difference between the *E. coli* and *Pseudomonas* RpsF modification systems is the presence and integral functional role of RimB. RimB is a protease that interacts directly with RimK_PS/PA_ and constrains the number of glutamate residues present on the C‐terminus of RpsF by targeted proteolysis.^10^, ^11^ Conversely, RimK_EC_ self‐limits the number of glutamates it adds to RpsF, negating the need for RimB. By comparing the divergent amino acid residues between the different RimK protein structures, we looked for clues as to the possible location of the RimB binding site.

**FIGURE 6:**
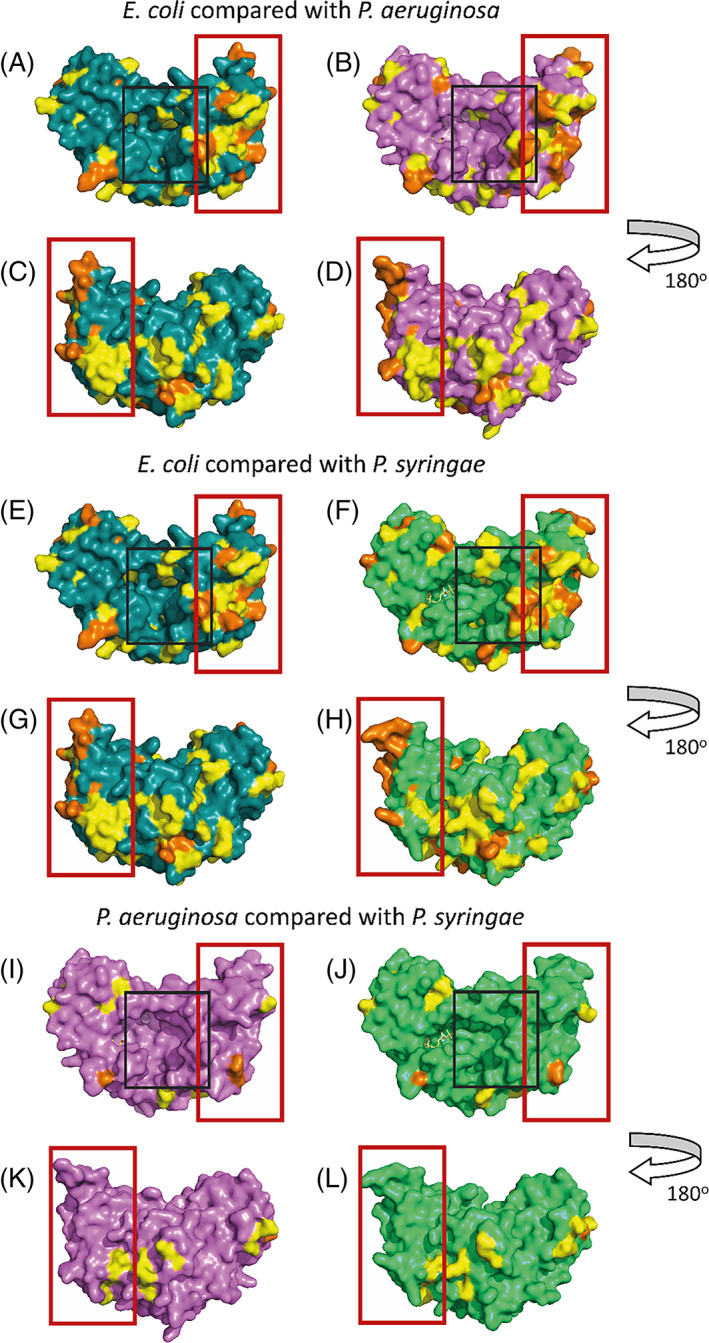
Amino acid sequence comparison between each of the RimK homologues points to a divergent region at the termini end of the protein. Amino acid sequence divergence was mapped onto the monomeric structure of RimK_PA_ (pink), RimK_EC_ (teal) and RimK_PS_ (green) with divergent residues shown in orange and conservative substitutions shown in yellow. RimK_EC_ (A and C) and RimK_PA_ (B and D), RimK_EC_ (E and G) and RimK_PS_ (F and H) and RimK_PA_ (I and K) and RimK_PS_ (J and L) are compared with divergent residues highlighted on both monomers. Monomers are shown at two orientations with ADP shown in light yellow and glutamate in gray. The glutamate binding site is highlighted with a gray box and the divergent terminal region is highlighted with a red box.

Comparing the primary amino acid sequences for RimK_EC_ and RimK_PA_, we observed a distinct area of divergence between the two structures, at the terminal end and on the external face of the monomeric protein (highlighted in Figure 6A–D). This divergent area is also present when comparing the amino acid structures of RimK_PS_ and RimK_EC_ (Figure 6E–H). Comparing the RimK_PA_ and RimK_PS_ proteins (Figure 6I), we saw comparatively few divergent residues, with only a small number of mostly conservative residue substitutions on the external faces of the protein. This sequence divergence highlights the RimK terminal region as a possible interaction site for RimB. It also could suggest that the terminal region of the protein may have an impact on the mechanistic differences seen between the *E. coli* and *Pseudomonas* proteins.

## 4 PREDICTING THE INTERACTION SURFACE BETWEEN RIMK_PA_ AND RIMB_PA_

The interaction between RimK and RimB in *P. fluorescens* SBW25 has been demonstrated biochemically in previous work from Little and colleagues.^10^, ^11^ RimB addition stimulates RimK ATPase activity in vitro, but also functions as a modulator of the poly‐α‐glutamate chain length on RpsF.^11^ Curiously, the interaction between RimB_PA_ and RimK_PA_ appears to differ considerably from RimK_PF/PS_ with its native RimB_PF/PS_. Contrary to the stimulation of RimK activity seen for *P. fluorescens* ^10^, ^11^ (or *P. syringae* [Figure 4B]), the ATPase activity of RimK_PA_ decreases as the concentration of RimB_PA_ increases (Figure 4C), suggesting that RimB interaction depresses RimK ATPase activity rather than stimulating it.

To gain additional insight into the potential mechanism of stimulation/suppression of RimK activity by RimB, we used the comparative structural analysis with RimK_EC_ (Figure 6) as the basis for modeling the potential RimB‐RimK interaction surface. The quaternary structure of RimK is defined by the interacting active sites in the central channel of the tetramer. Based on this, it seems likely that the binding site for RimB lies on the exposed RimK monomer surfaces in the tetrameric conformation.

**FIGURE 7:**
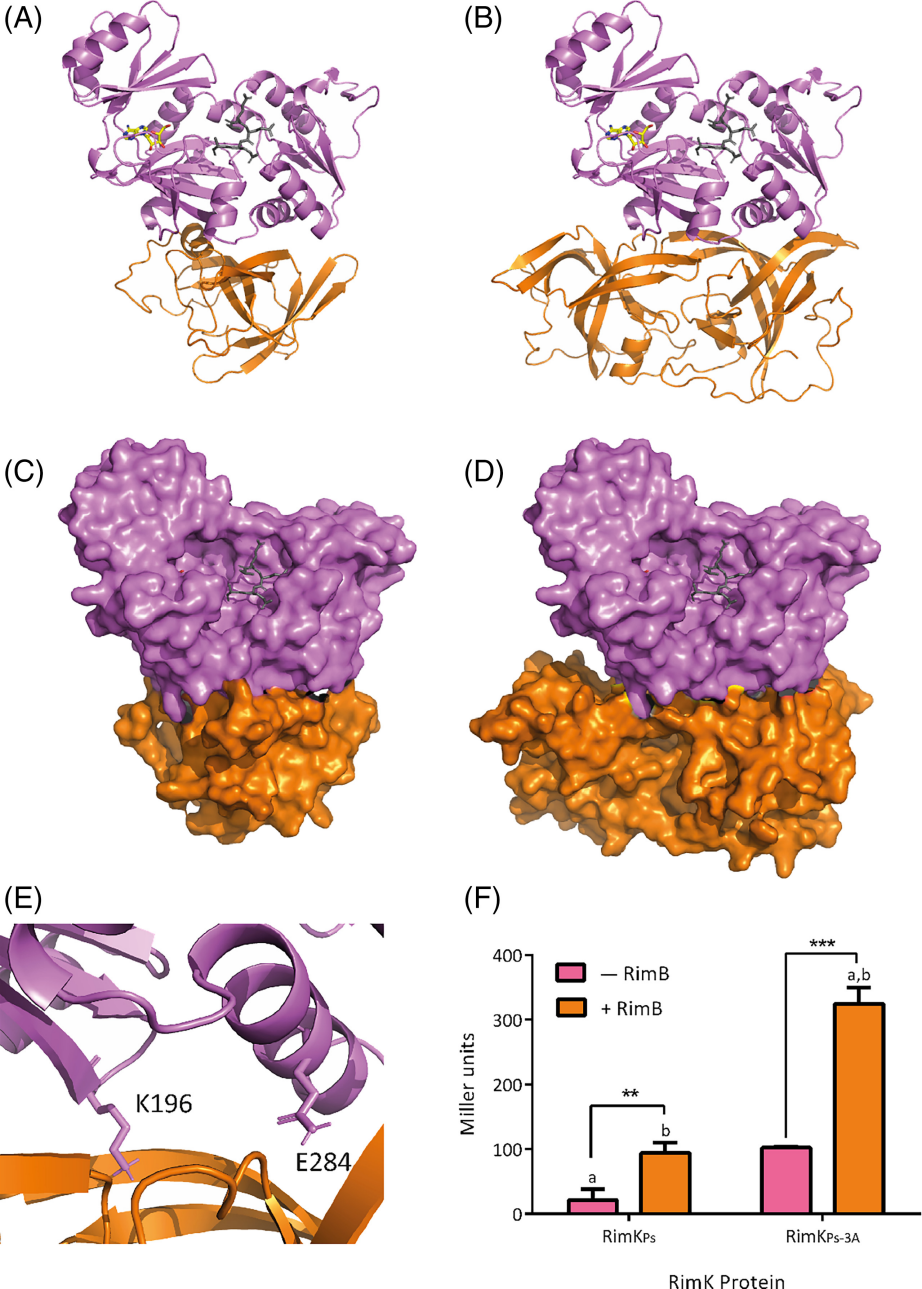
Cartoon representations of RimB_PA_ modeled onto the monomer of RimK_PA._ HADDOCK docking modeling of AlphaFold modeled RimB as both a monomer (A and C) and dimer (B and D) docked onto a divergent region between RimK_PA_ and RimK_EC._ The model is shown with both RimK as a cartoon (A and B) and as a surface (C and D) with the ADP residues shown in yellow and the glutamates as gray sticks with RimB shown in orange. (E) Close up image of K196 and E284 residues on RimK and (F) ATPase results for RimK_Ps_ and triple mutant (K196A, E284A and N294A) RimK_Ps‐3A._ A Two‐way ANOVA indicated significance for both RimK (*p* < .001) and RimB addition (*p* < .001), with multiple comparisons showing difference between conditions (indicated by letters, *p* < .01). Individual *t*‐tests were performed to compare ±RimB conditions and significance is indicated (*p* < .01 **, *p* < .001 ***).

Despite our best efforts, RimB proved resistant to crystallization. Therefore, in order to model this interaction, we created three dimensional models of monomeric and dimeric RimB_PA_ using AlphaFold^28^ (as many proteases are predicted to act as dimers). Using this AlphaFold model, RimB_PA_ was docked onto the sequence‐divergent region of RimK_PA_ (Figure 6) using HADDOCK^23^ to predict potential interaction sites between RimK and RimB. Docking modeling for both the monomer and the dimer of RimB gave similar RimK interaction sites (Figure 7A–D) and suggested that the interaction surface for RimB_PA_ lies between B5 and B6 and the H6 and H7 region of the monomeric RimK_PA_. This binding interaction was seen with both the monomeric and the dimeric RimB structures, even though they interact in different orientations.

The proposed RimK‐RimB docking model was tested by the creation of a triple mutation within RimK at the predicted binding interface. Residues K196, E284 and N294 of RimK were all altered to alanine (RimK_PS‐3A_, Figure 7E). We predicted that these mutations would disrupt the RimK‐RimB interaction and alter the RimK response to RimB addition. Interestingly, the triple mutation showed a large and unexpected increase in RimK ATPase activity, that could be further stimulated by RimB addition (Figure 7F). While this seems to exclude residues K196, E284, and N294 from contributing directly to RimB interaction, our results are consistent with this protein region playing a key role in the control of RimK ATPase activity.

As RimK has been shown to exist in a tetrameric state it was important to validate whether our docking models would still be sterically valid when mapped onto a tetramer of RimK_PA._ Both the monomers and dimers of RimB sit on the extremities of each RimK monomeric unit and the oligomerization does not compromise either RimB binding or tetramer formation (Figure S2). Although our docking modeling is not as accurate as solving a high‐resolution structure, given the experimental intractability of RimB it gives some valuable initial insights into how RimK‐RimB interactions may occur and informs further work to unpick the structure–function relationship of this system.

## 5 DISCUSSION

In this paper we have solved and analyzed the structures of two new RimK proteins from *Pseudomonas* spp. Our results show that although structurally similar, there are distinct differences between the *E. coli* and *Pseudomonas* RimK proteins that allow some insights into their functional differences. Although biochemically RimK_PS/PF_ and RimK_EC_ are well understood, the mechanism of RimK_PA_ function has remained elusive. RimK plays an important role in virulence in both *P. aeruginosa* and *P. syringae*,^10^ so it is important to understand the similarities and differences of RimK activity and regulation between these different species. The resolution of the proteins in a tetrameric state supports the recent modeling by Grenga, Little and colleagues.^11^

The high degree of structural similarity between the different RimK proteins is curious as they are all quite distinct biochemically, with markedly different ATPase activity rates and relationships to their interaction partners. When compared with RimK_EC_ the residues that form the ATP binding site (E178, L141, N213, and S212) are both conserved between RimK_EC_ and RimK_PS/PA_ as well as being in a similar spatial orientation in all three *Pseudomonas* proteins. Therefore, the discrepancy between the biochemical behavior of the different RimK proteins is likely to stem from small but potentially significant differences in protein tertiary structure.

**FIGURE 8:**
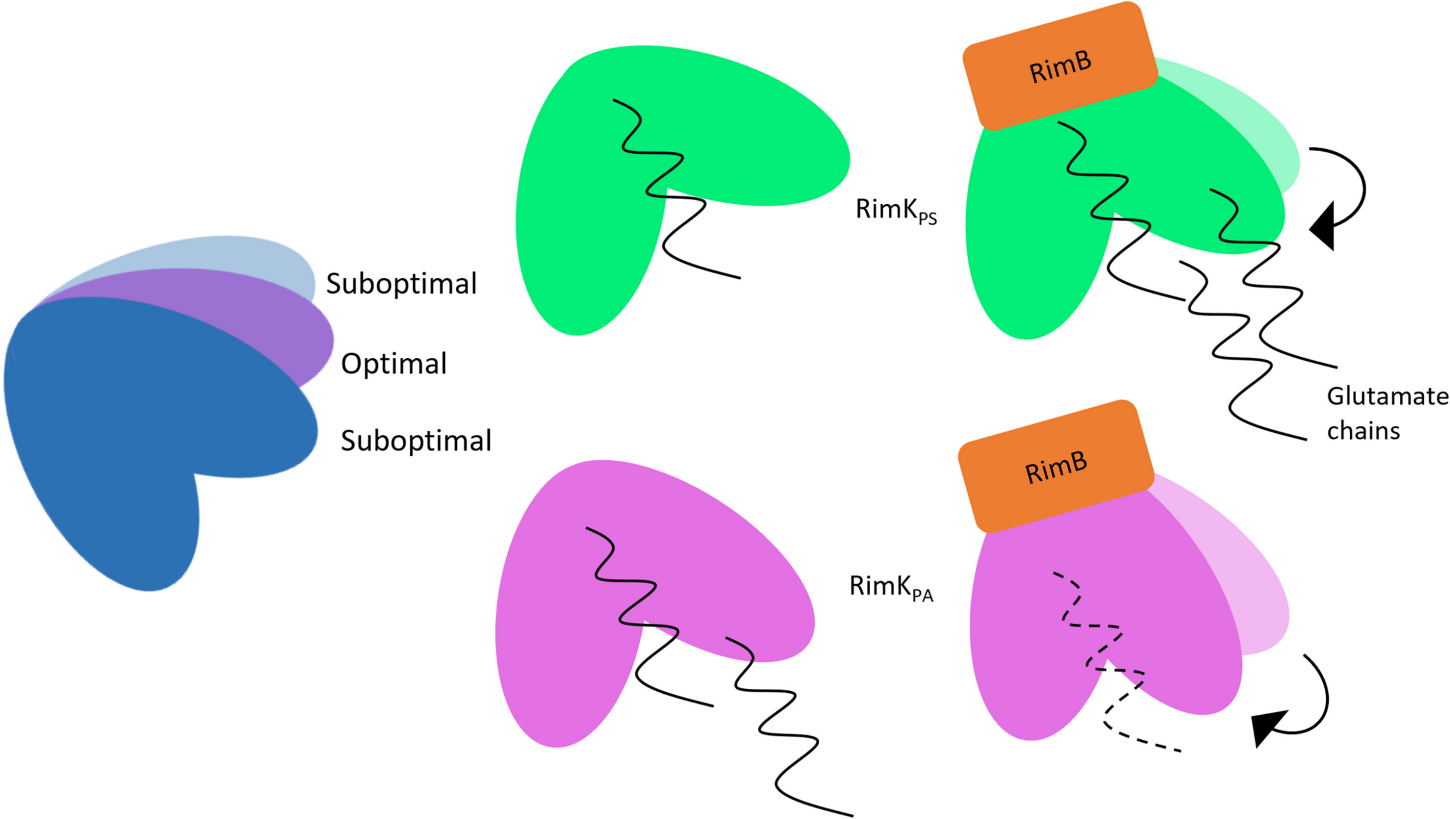
Schematic diagram of proposed changes in the RimK active site triggered by interaction with RimB. Cartoon representation of RimK showing the relative openness of the active site for RimK_PS_ (green) and RimK_PA_ (pink) and proposed effects on the rate of poly‐E glutamate production (black). The binding of RimB is suggested to reduce the openness of the active site of RimK and this is predicted in turn to affect enzyme activity.

A detailed comparison of the similarities and differences between the two structures enabled us to propose a model for RimB modulation of RimK activity. RimK_PA_ and RimK_PS_ only show a small difference in structure in the ATP/ADP binding site. However, their *V*_max_ values were significantly different. Moreover, the reduction in ATPase activity with the addition of RimB_PA_ to RimK_PA_ (as opposed to the stimulation of activity seen with RimK_PS_) suggests that there are substantive, mechanistic differences between the two proteins that manifest when an interaction with RimB occurs, confirmed by the creation of a chimeric protein. The more closed active site conformation of RimK_EC_ compared to RimK_PA_, itself presenting a more closed conformation than RimK_PS_, provides a possible explanation for the differences seen in *V*_max_ and the enzymatic changes seen upon RimB binding. It is plausible that the interaction between RimB and RimK results in a more “closed” RimK conformation, resulting in the ATP/glutamate binding sites being brought closer together (Figure 8). In this model, RimB interaction with RimK_PA_ results in the over‐constriction of the ATP and glutamate binding sites of the protein, therefore reducing the glutamate ligation efficiency in the absence of RpsF. Conversely, closing the naturally under‐constricted active site of RimK_PS_ through RimB interaction would generate the optimal distance between ATP and glutamate, resulting in an increased efficiency of ATPase activity and glutamate ligation. Although the creation of Poly‐E chains requires ATPase activity it has been seen that only a basal amount of this is required to modify RpsF when all protein partners are present, further indicating the complexities of this system in vivo.

For the first time, we have been able to resolve glutamate within the active site of a RimK protein. Although the glutamate binding residues in the active site had been predicted in RimK_EC_, (poly)glutamate had not been resolved in the active sites of the structures produced so far. Our model of the glutamate‐bound active site highlights the broad overall conservation of this motif between the various resolved RimK structures. Furthermore, our data both confirms and expands upon the active site predictions from previous *E. coli* models, identifying several conserved, coordinating residues for poly‐glutamate. Structural comparisons highlight a distinct difference in the orientation of active site residues between RimK_PA/PS_ and RimK_EC_, which leads to a more closed formation of the active site in RimK_EC_ when compared to the *Pseudomonas* RimK proteins. Previous work has shown that RimK_EC_ is able to self‐regulate the number of glutamates added to RpsF, whereas *Pseudomonas* proteins rely on the proteolytic activity of RimB to determine the number of glutamates added to RpsF. It seems likely that these active site differences may aid in this self‐limitation by RimK_EC_, possibly by providing a steric hindrance to further chain elongation after the attachment of a certain number of glutamate residues.

The interaction between RimK and RimB is central to the distinct differences in biochemistry and function observed between RimK_EC_, RimK_PA_ and RimK_PS_, with a few relatively small structural differences in RimK being the key to this difference. We propose that steric constraints in the glutamate binding site, and its relative proximity to the ATPase site define both the extent of glutamate ligation, which may be increased or decreased by RimB interaction, and the ability of RimK to restrict the length of poly‐glutamate chains. If RimB binding to RimK_PA/PS_ leads to their binding sites more closely resembling that of RimK_EC_, this could potentially both modulate the RimK *V*_max_ and limit poly‐glutamate chain length, although this second function would be masked by the poly‐glutamate protease activity of RimB. Future research, into the biochemical activity of RimB and the mechanics of its interaction with RimK, will address this possibility.

A central unresolved question concerns the mechanism of action of cdG. This dinucleotide signaling molecule interacts specifically with both *Pseudomonas* RimK proteins and stimulates their ATPase activity^10^ (Figure S1) with the activity of RimK_PF_ (and presumably RimK_PS_) also controlled by the cdG phosphodiesterase RimA. However, we could not resolve bound cdG in any of our crystal structures. Given their high primary and tertiary similarity, it is reasonable to speculate that the cdG binding site and activation mechanism are likely conserved between the *Pseudomonas* RimK variants. Likewise, it is unlikely that cdG functions in the same way as RimB binding, as otherwise we might expect cdG to suppress, rather than stimulate RimK_PA_. CdG binding sites are highly diverse, typically comprising a few correctly placed arginine, aspartate, and glutamate residues, and as such are resistant to bioinformatic prediction.^29^ Our results suggest that small structural differences within the protein can be sufficient to account for differential activity, therefore, the complete picture of the cdG binding site, and the mechanism of RimK stimulation necessarily await future structural solution.

In this paper we used a combination of AlphaFold models of RimB, RimK primary structure divergence and docking modeling to predict a potential interaction site for RimB binding to RimK_PA_. This modeling suggested plausible RimB interactions with RimK as either a monomer or a dimer and enabled us to propose a mechanism of RimK regulation in the absence of either a RimB or a RimBK co‐crystal structure and was supported by the biochemical data. Looking forward, the combination of structural biology, comparative sequence analysis and computational modeling we use in this study has significant potential to inform our understanding of complex protein–protein interactions, especially for systems where one or more component is resistant to experimental interrogation.

## FUNDING INFORMATION

Catriona Thompson: UK Research and Innovation | Biotechnology and Biological Sciences Research Council (BBSRC) Responsive Mode Grant BB/R018154/1 to Jacob G. Malone; Jacob Malone, Richard Little: UK Research and Innovation | Biotechnology and Biological Sciences Research Council (BBSRC) Institute Strategic Program Grant BBS/E/J/000PR9797 to the John Innes Centre; David Lawson, Clare Stevenson: UK Research and Innovation | Biotechnology and Biological Sciences Research Council (BBSRC) Institute Strategic Program Grant BBS/E/J/000PR9790 to the John Innes Centre.

## CONFLICT OF INTEREST

There are no conflicts of interest to declare.

### PEER REVIEW

The peer review history for this article is available at https://publons.com/publon/10.1002/prot.26429.

## Supporting information


**Figure S1** Cyclic‐di‐GMP stimulates the ATPase activity of RimK_PS_ and RimK_PA_ a) ATPase activity of 1.5 μM RimK_PS_ in the absence (solid line; *V*_max_ = 201.3 nmol/min/mg, *K*_m_ = 1.85 mM) or presence (dashed line; *V*_max_ = 264.6 nmol/min/mg, *K*_m_ = 0.84 mM) of 25 μM Cyclic‐di‐GMP. b) ATPase activity of 2.5 μM RimK_PA_ in the absence (solid line; *V*_max_ = 73.1 nmol/min/mg, *K*_m_ = 2.1 mM) or presence (dashed line; *V*_max_ = 115.8 nmol/min/mg, *K*_m_ = 2.2 mM) of 25 μM Cyclic‐di‐GMP or in the presence (dotted line; *V*_max_ = 75.6 nmol/min/mg, *K*_m_ = 2.4 mM) of 25 μM Cyclic‐di‐AMP. Individual points represent absolute data points to which a non‐linear regression fit has been applied.

**Figure S2** Cartoon representations of RimB_PA_ modeled onto the Tetramer of RimK_PA_. HADDOCK docking modeling of AlphaFold modeled RimB_PA_ as both a monomer (a and c) and dimer (b and d) docked onto a region of RimK_PA_ that was divergent from RimK_EC_. The model is shown from two angles with each of the RimK chains shown as a different shade of blue and each of the RimB chains shown as a different shade of red

**Table S1:** Strains and Plasmids.

## Data Availability

The data that support the findings of this study are openly available in the protein data bank under accession numbers 7QYR and 7QYS.
