# Supplementary material for: Structural insights into the mechanism of adaptive ribosomal modification by Pseudomonas RimK
Source: Proteins. 2022 Oct 6;91(3):300–14. doi: 10.1002/prot.26429 (PMC10092738; doi:10.1002/prot.26429)
Supplement: Supplementary file 1 — Figure S1 Cyclic‐di‐GMP stimulates the ATPase activity of RimKPS and RimKPA a) ATPase activity of 1.5 μM RimKPS in the absence (solid line; V max = 201.3 nmol/min/mg, K m = 1.85 mM) or presence (dashed line; V max = 264.6 nmol/min/mg, K m = 0.84 mM) of 25 μM Cyclic‐di‐GMP. b) ATPase activity of 2.5 μM RimKPA in the absence (solid line; V max = 73.1 nmol/min/mg, K m = 2.1 mM) or presence (dashed line; V max = 115.8 nmol/min/mg, K m = 2.2 mM) of 25 μM Cyclic‐di‐GMP or in the presence (dotted line; V max = 75.6 nmol/min/mg, K m = 2.4 mM) of 25 μM Cyclic‐di‐AMP. Individual points represent absolute data points to which a non‐linear regression fit has been applied. [file PROT-91-300-s002.pdf]

a

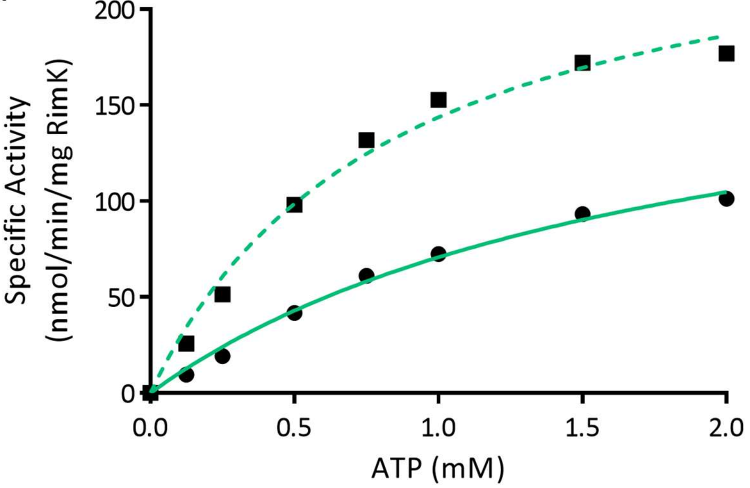

b

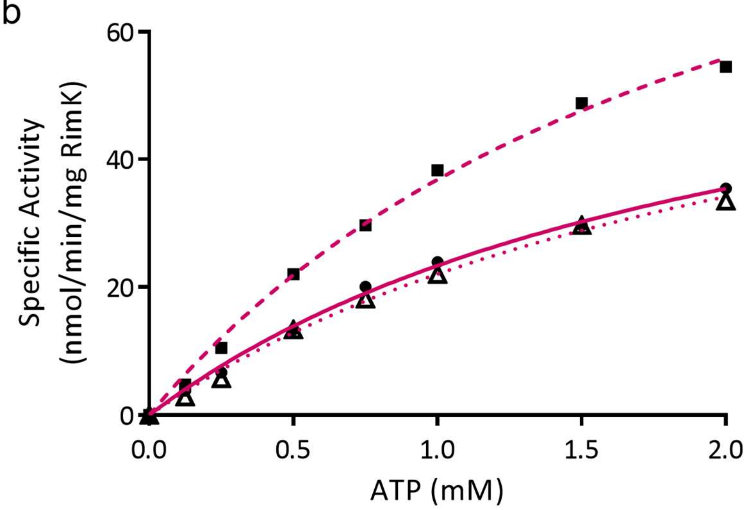

Figure S1: Cyclic-di-GMP stimulates the ATPase activity of RimK$_{PS}$ and RimK$_{PA}$ a) ATPase activity of 1.5 µM RimK$_{PS}$ in the absence (solid line; Vmax = 201.3 nmol/min/mg, Km = 1.85 mM) or presence (dashed line; Vmax = 264.6 nmol/min/mg, Km = 0.84 mM) of 25 µM Cyclic-di-GMP. b) ATPase activity of 2.5 µM RimK$_{PA}$ in the absence (solid line; Vmax = 73.1 nmol/min/mg, Km = 2.1 mM) or presence (dashed line; Vmax = 115.8 nmol/min/mg, Km = 2.2 mM) of 25 µM Cyclic-di-GMP or in the presence (dotted line; Vmax = 75.6 nmol/min/mg, Km = 2.4 mM) of 25 µM Cyclic-di-AMP. Individual points represent absolute data points to which a non-linear regression fit has been applied.
