# Supplementary material for: Structural insights into the mechanism of adaptive ribosomal modification by Pseudomonas RimK
Source: Proteins. 2022 Oct 6;91(3):300–14. doi: 10.1002/prot.26429 (PMC10092738; doi:10.1002/prot.26429)
Supplement: Supplementary file 2 — Figure S2 Cartoon representations of RimBPA modeled onto the Tetramer of RimKPA. HADDOCK docking modeling of AlphaFold modeled RimBPA as both a monomer (a and c) and dimer (b and d) docked onto a region of RimKPA that was divergent from RimKEC. The model is shown from two angles with each of the RimK chains shown as a different shade of blue and each of the RimB chains shown as a different shade of red [file PROT-91-300-s003.pdf]

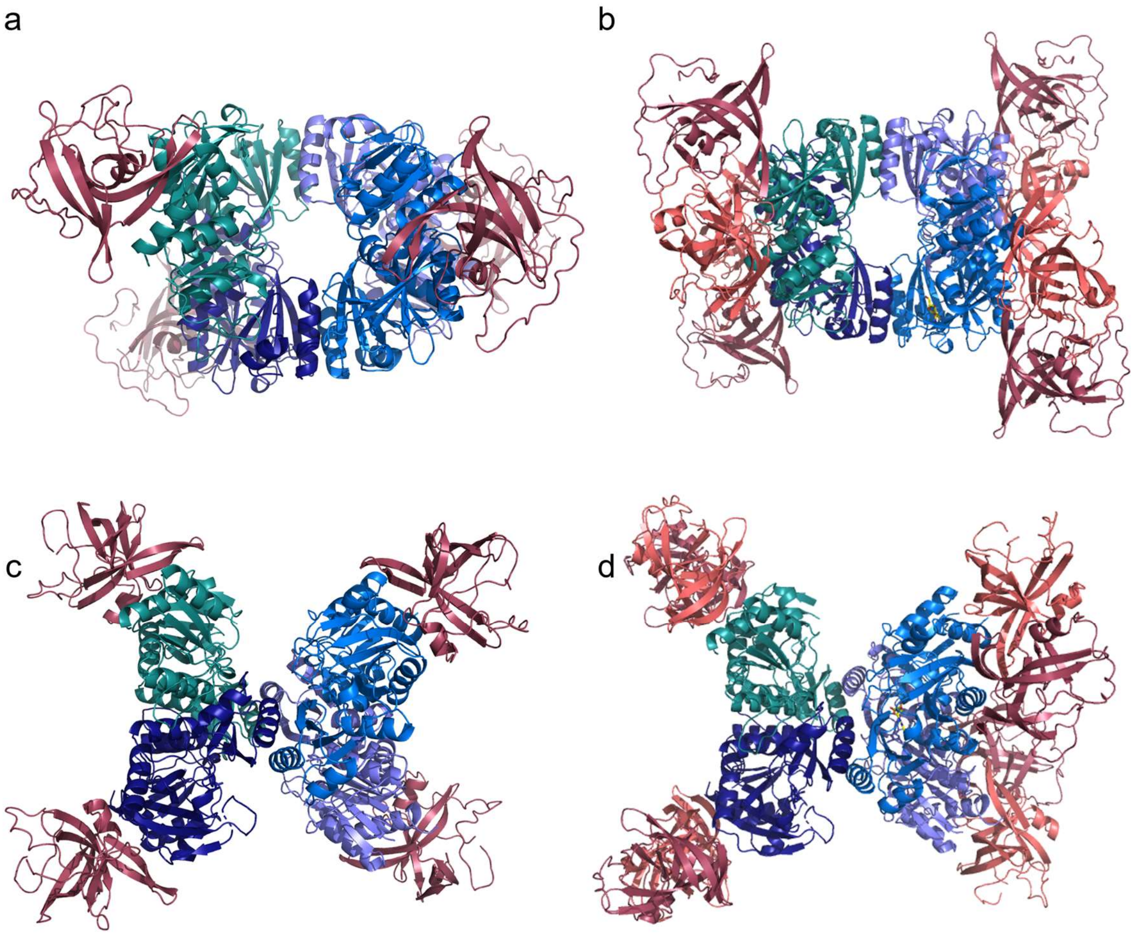

Supplementary figure 2: Cartoon representations of $RimB_{PA}$ modelled onto the Tetramer of $RimK_{PA}$. HADDOCK docking modelling of AlphaFold modelled $RimB_{PA}$ as both a monomer (a and c) and dimer (b and d) docked onto a region of $RimK_{PA}$ that was divergent from $RimK_{EC}$. The model is shown from two angles with each of the RimK chains shown as a different shade of blue and each of the RimB chains shown as a different shade of red
