# Supplementary material for: Structural insights into the mechanism of adaptive ribosomal modification by Pseudomonas RimK
Source: Proteins. 2022 Oct 6;91(3):300–14. doi: 10.1002/prot.26429 (PMC10092738; doi:10.1002/prot.26429)
Supplement: Supplementary file 3 — Table S1: Strains and Plasmids. [file PROT-91-300-s001.docx]

Supplementary table 1: Strains and Plasmids

| Strain | Description | Reference |
| --- | --- | --- |
| *E. coli* |  |  |
| DH5α | *endA*1, *hsdR*17(r_K_-m_K_+), *supE*44, *recA*1, *gyrA* (Nal^r^), *relA*1, Δ(*lacIZYA-argF*) U169, *deoR*, Φ80*dlacΔ(lacZ)*M15 | **Woodcock *et al*, 1989** ^28^ |
| BL21-(DE3) pLysS | Sm^R^, k12 *recF143 lacI^q^ lacZΔ.M15, xylA,* pLysS | Novagen |
| Plasmids |  |  |
| pETM11-*rimB* | pET*Nde*M-11 with SBW25 *rimB* as *NdeI-XhoI* fragment | **Little *et al*, 2016** ^9^ |
| pET42b(+)-*rimK* | pET42b(+) with SBW25 *rimK* as *NdeI-XhoI* fragment | **Little *et al*, 2016** ^9^ |
| pDCrimK29a | pET29a(+) with DC3000 *rimK* as *NdeI-XhoI* fragment | This Laboratory |
| pPArimK29a | pET29a(+) with PAO1 *rimK* as *NdeI-HindIII* fragment | This Laboratory |
| pETM11-rimB | pET*Nde*M-11 with SBW25 *rimB* as *Nde*I-*Xho*I fragment | **Grenga *et al*, 2020** ^10^ |
| pET*Nde*M-11 | Km^R^, purification vector, N-terminal His_6_-tag | **Little, Salinas…, 2011** |
| Primers | **Sequence** | **Description** |
| 0261NdeFor | CAGAAGCCATATGAAGATTGCTGTGCTGTCG | *PFLU0261* (SBW25 *rimK*) purification, forward primer |
| 0261XhoRev | GGTACTCGAGGCCCTTGCCTTTGGTCCGAGTC | *PFLU0261* (SBW25 *rimK*) purification, reverse primer |
| DCRimKNdeFor | CAGAAGCCATATGAAGATCGCTGTGCTTTC | PSPTO_0234 (DC3000 *rimK*)  Purification, forward primer |
| DCRimKXhoRev1 | GGTACTCGAGGCCTTTTCCCTTGGTGCG | PSPTO_0234 (DC3000 *rimK*)  Purification, reverse primer |
| PARimKNdeFor | CAGAAGCCATATGAAAATCGCCGTGCTGTC | PA5197 (PAO1 *rimK*)  Purification, forward primer |
| PARimKHindRev1 | GGTAAAGCTTCCCCTTTCCCTTCGTCC | PA5197 (PAO1 *rimK*)  Purification, reverse primer |
| 0262NdeFor | CAGAAGCCATATGAAGACATTTGACCATTTG | PFLU0262 (SBW25 *rimB*) purification, forward primer |
| 0262XhoRev2 | GGTACTCGAGTCATGCAGCACCTGGGGC | PFLU0262 (SBW25 *rimB*) purification, reverse primer |
